# Risk Factors for Rectal Cancer Recurrence after Local Excision of T1 Lesions from a Decade-Long Multicenter Retrospective Study

**DOI:** 10.3390/jcm13144139

**Published:** 2024-07-16

**Authors:** Yaron Rudnicki, Nitzan Goldberg, Nir Horesh, Assaf Harbi, Barak Lubianiker, Eraan Green, Guy Raveh, Moran Slavin, Lior Segev, Haim Gilshtein, Alexander Barenboim, Nir Wasserberg, Marat Khaikin, Hagit Tulchinsky, Nidal Issa, Daniel Duek, Shmuel Avital, Ian White

**Affiliations:** 1 Department of Surgery, Meir Medical Center, Faculty of Medicine, Tel Aviv University, Tel Aviv 6997801, Israel; 2 Department of General Surgery B and Organ Transplantation, Sheba Medical Center, Faculty of Medicine, Tel Aviv University, Tel Aviv 6997801, Israel; 3 Department of General Surgery, Rambam Health Care Campus, Rappaport Faculty of Medicine, Technion-Israel Institute of Technology, Haifa 3109601, Israel; 4 Department of Surgery, Rabin Medical Center—Hasharon Hospital, Faculty of Medicine, Tel-Aviv University, Tel Aviv 6997801, Israel; 5 Department of Surgery, Tel Aviv Sourasky Medical Center, Sackler Faculty of Medicine, Tel Aviv University, Tel Aviv 6997801, Israel; 6 Department of Surgery, Rabin Medical Center—Beilinson Hospital, Faculty of Medicine, Tel Aviv University, Tel Aviv 6997801, Israel

**Keywords:** early rectal cancer, t1 rectal cancer, local excision, rectal cancer recurrence

## Abstract

**Background:** Local surgical excision of T1 rectal adenocarcinoma is a well-established approach. Yet, there are still open questions regarding the recurrence rates and its risk factors. **Methods:** A retrospective multicenter study including all patients who underwent local excision of early rectal cancer with an open or MIS approach and had a T1 lesion from 2010 to 2020 in six academic centers. Data included demographics, preoperative studies, surgical findings, postoperative outcomes, and local and systemic recurrence. A univariable and multivariable logistic regression analysis was performed to identify risk factors for recurrence. **Results:** Overall, 274 patients underwent local excision of rectal lesions. Of them, 97 (35.4%) patients with a T1 lesion were included in the cohort. The mean age was 69 ± 10.5 years, and 42 (43.3%) were female. The mean distance of the lesions from the anal verge was 7.8 ± 3.2 cm, and the average tumor size was 2.7 ± 1.6 cm. Eighty-two patients (85%) had a full-thickness resection. Eight patients (8%) had postoperative complications. Kikuchi classification of submucosal (SM) involvement was reported in 29 (30%) patients. Twelve patients had SM1, two SM2, and fifteen SM3. Following pathology, 24 patients (24.7%) returned for additional surgery or treatment. The overall recurrence rate was 14.4% (14 patients), with 11 patients having a local recurrence and 6 having a systemic metastatic recurrence, 3 of which had both. The mean time for recurrence was 2.78 ± 2.8 years and the overall mortality rate was 11%. On univariable and multivariable logistic regression analysis of recurrence vs. non-recurrence groups, the strongest and most significant association and possible risk factors for recurrence were larger lesions (4.3 vs. 2.5 cm, *p* < 0.001) with an OR of 6.67 (CI—1.82–24.36), especially for tumors larger than 3.5 cm, mucinous histology (14.3% vs. 1.2%, *p* = 0.004, OR of 14.02, CI—1.13–173.85), and involved margins (41.7% vs. 16.2%, *p* = 0.003, OR of 9.59, CI—2.14–43.07). The open transanal excision (TAE) approach was also identified as a possible significant risk factor in univariant analysis, while SM3 level penetration showed only a trend. **Conclusion:** Surgical local excision of T1 rectal malignancy is a safe and viable option. Still, one in four patients received additional treatment. There is an almost 15% chance for recurrence, especially in large tumors, mucinous histology, or involved margin cases. These high-risk patients might warrant additional intervention and stricter surveillance protocols.

## 1. Introduction

Early rectal cancer, specifically lesions that have invaded only into the submucosal layer, without nodal or distant metastasis, are considered T1 lesions and represent a better overall prognosis [1]. Although total mesorectal excision (TME) offers the best oncological prognosis, it has certain inherited morbidities and possible major complications, whereas performing an organ preservation approach with a local excision (LE) on the appropriate lesion might offer acceptable long-term outcomes with a much lower morbidity burden and fewer complications [2]. Local excision of a rectal mass can be performed in a few different approaches and platforms, with a known superiority for minimally invasive techniques such as transanal endoscopic microsurgery (TEM) or transanal minimally invasive surgery (TAMIS) that allow for better quality specimens, access to higher rectal lesions, and less postoperative complications [3]. Full-thickness resection is important for optimal pathological assessment [4]. The key factors in choosing the appropriate rectal lesion for LE are based on the accurate preoperative assessment of the lesion, mainly on the proper characterization on endoscopy and focused imaging of the rectal wall invasion. 

Reports on local recurrence rates following an LE of a T1 tumor range from 4.1% to 20.5% [5,6,7,8,9]. Yet, based on the limited literature, when T1 lesions are broken down by the level of submucosal (SM) invasion, according to the Kikuchi classification [10], the rate of local recurrence for T1 SM3 tumors can be as high as 30% [11] and the distant metastasis as high as 35% [12]. Other reported risk factors for recurrence are non-TEM/TAMIS resection methods, high tumor grade, perineural or lymphovascular invasion, and large tumors [8,13,14]. This study aimed to assess the outcome of local excision of a T1 rectal cancer, assess the recurrence rate, and identify possible risk factors for recurrence. 

## 2. Materials and Methods

A retrospective-cohort multicenter study included all patients who underwent local excision of rectal lesions from 2010 to 2020 from six academic medical centers in Israel. A colon and rectal surgery specialist ran the study and cases in each center. All cases of transanal excision were collected in the initial database. After a review of the pathology reports, only cases with T1 rectal cancer were included in this current study. The operative platforms used for resection were transanal endoscopic microsurgery (TEM), transanal minimally invasive surgery (TAMIS), and a standard transanal excision (TAE). Patients’ data included demographic characteristics (age, gender, body mass index (BMI), comorbidities, American Society of Anesthesiology (ASA) score), preoperative studies including flexible sigmoidoscopy, colonoscopy, or rigid proctoscopy with rectal lesion biopsy, abdominal and pelvic CT, transrectal ultrasound (TRUS), and pelvic magnetic resonance imaging (MRI). Surgical data such as surgical approach and findings, length of stay, postoperative complications, morbidity and mortality, and postoperative outcome were collected from operative reports and follow-up charts. Detailed pathological reports were reviewed for lesions’ histology and characteristics, and outpatient visits and follow-up charts were monitored for malignant recurrence and following treatment for recurrence. The Clavien–Dindo classification of surgical complications scale was used to classify postoperative complications [15]. Analysis of outcome by submucosal (SM) involvement was performed by grouping SM1 and SM2 together and comparing them to SM3, as multiple studies showed similar outcomes for SM1 and SM2 [16,17]. 

Statistical analyses were performed using IBM SPSS software version 25.0. Univariable analysis was used to compare patients with local recurrence and patients without local recurrence regarding the background characteristics, preoperational evaluation, pathological results, and postoperational events. Dichotomous and categorical variables were compared using the Chi-square test and are presented as number of cases and percentage. Continuous variables were compared using the independent T-test if they were normally distributed or the Mann–Whitney test if they were not normally distributed. Normal distribution was tested using the Kolmogorov–Smirnov test. Continuous variables are presented as mean and standard deviation. Significant variables were tested in a multivariable logistic regression to assess the association between specific pathological characteristics of the sample and local recurrence. All logistic regression models were adjusted to relevant background characteristics. A *p*-value <0.05 was considered significant. All analyses were two-sided. Each of the six participating centers’ local institutional review boards approved the study.

## 3. Results

Two hundred and seventy-four patients who underwent local excision of rectal lesions were included in the database. Of those, only 97 patients (35.4%) had a pathology of rectal adenocarcinoma that penetrated the rectal submucosa and was considered a T1 lesion. The mean age at diagnosis was 68 ± 11 years. Forty-two patients (43%) were females, and the average BMI was 28 ± 4.8 kg/m². A total of 80 patients (82%) were found to have one comorbidity or more, including diabetes mellitus, hypertension, chronic respiratory, cardiac, or liver disease, or chronic autoimmune condition, and 29 (36.7%) had an American Society of Anesthesiologists score (ASA score) of three or above (Table 1).

### 3.1. Preoperative Assessment 

Endoscopic preoperative assessment of the lesions showed lesions with an average size of 3.5 ± 3.3 cm at a mean distance of 7.6 ± 3 cm from the anal verge on rectoscopy. Following an initial abdominal and pelvic CT scan, further rectal imaging was performed to assess the level of penetration. A transrectal ultrasound (TRUS) was performed on 57 patients (59%), and a pelvic MRI was performed for only 8 patients (8%), 7 of whom had also undergone a TRUS. Out of the seven patients who had both TRUS and MRI, there were only four patients for whom the two imaging modalities agreed regarding the penetration level. 

### 3.2. Surgical Approach, Postoperative Complications, and Pathological Findings 

A total of 61 patients (63%) had their rectal lesion resected via the TEM platform, 29 patients (30%) underwent laparoscopic TAMIS, and 7 patients (7%) had a standard TAE. The average lesion size reported was 2.7 ± 1.6 cm. The mean distance of the lesions from the anal verge was 7.8 ± 3.2 cm, similar to the rectoscopy reports. Full-thickness resection was achieved in 82 (84.5%) cases, 11 (11.3%) cases had a partial-thickness resection (any resection that did not fully reach the mesorectum), and 4 (4.1%) patients had the lesion resected in more than one piece (piecemeal resection). The rates of involved margins, depth of resection, and complications by platform are described in Table 2. In four cases (4%), a laparoscopy was added to the transanal approach to rule out intraperitoneal violation, no attempts were made to close the defect from the abdominal side, and intraabdominal drains were left in three patients with no major complications. 

Postoperative complications were seen in eight patients (8.2%). Three patients had postoperative bleeding, and three patients had infectious complications in the form of one confirmed leak, one pelvic abscess, and one surgical site infection. These three patients were taken back to surgery for lavage and drainage and are categorized with a Clavien–Dindo complication score of IIIb. Two patients had a urinary infection and respiratory complications with a Clavien–Dindo complication score of one.

Upon reviewing the pathological reports, there were 87 (89.2%) patients with clear margins, 13 of them with clear margins but were less than 3 millimeters. Ten patients (10.8%) had positive margins. The pathological grading was well-differentiated adenocarcinoma in 50 patients (52.1%), moderately differentiated adenocarcinoma in 33 patients (34.4%), 5 patients had a poorly differentiated adenocarcinoma (5.2%), 5 patients had adenocarcinoma not otherwise specified (NOS) (5.2%), and 3 patients (3.1%) had mucinous adenocarcinoma. Lymphovascular invasion was present in 9 patients (9.4%), tumor budding was seen in 12 patients (12.4%), and no perineural invasion was seen. 

Following surgical pathology, 24 patients (24.7%) had additional treatment added. Seven patients (7.2%) went back for an abdominoperineal resection (APR), seven patients (7.2%) had low anterior resection (LAR), six patients (6.2%) went back for another local excision, and four patients (4.1%) had added adjuvant therapy in the form radiotherapy, chemotherapy, or immunotherapy. Upon further analysis, out of the 14 patients that were taken back for an APR or an LAR, 5 had tumors larger than 3.5 cm, only 3 had involved margins, 4 had lymphovascular invasion, and 3 had tumor budding. Out of the six patients that had a re-do local excision, one had a tumor larger than 3.5 cm, three had involved margins, and none had poor histological markers. Of the four patients that received adjuvant therapy, two had tumors larger than 3.5 cm, one had involved margins, one had a mucinous adenocarcinoma, and one had tumor budding (Table 3).

### 3.3. Malignant Recurrence

The overall recurrence rate was 14.4% (14 patients). Eleven patients (11.3%) had a local recurrence in the rectum, and six patients (5.2%) had a systemic recurrence with metastatic disease, three of which had both local and metastatic recurrence. The mean time interval from initial local excision to recurrence was 1.1 ± 0.6 years. Following recurrence, two patients underwent an abdominoperineal resection (APR), one patient had a low anterior resection (LAR), four patients had another local excision from the rectum, two patients had only chemotherapy, three patients had a combination of radio-chemotherapy and surgery, and two patients had no treatment (Table 1). The overall mortality rate was 11 patients (11%) at a time interval of 7.8 ± 6.4 years. The overall mean follow-up time was 6.3 ± 4.7 years. A total of 5 (21%) out of the 24 patients who had an added treatment after the initial LE, either a formal rectal resection, another LE, or adjuvant therapy, also had a rectal cancer recurrence (Table 3). 

### 3.4. Sub-Analysis of Submucosal Involvement and Association with Recurrence and Possible Risk Factors

The Kikuchi classification of submucosal (SM) involvement was reported in only 29 patients (30%) out of the initial 97. A total of 12 patients were characterized as SM1, 2 as SM2, and 15 as SM3. The SM3 lesions were larger than the SM1 + 2 (3.5 ± 1.7 vs. 2.4 ± 1.2 cm). Three patients (20%) with SM3 had a recurrence, two local, and one systemic recurrence. Only one SM2 patient had a local recurrence, and there was none in the SM1 group (Table 4).

### 3.5. Univariable and Multivariable Logistic Regression Analysis Identifying Possible Risk Factors for Recurrence

A comparison between patients with a local or systemic recurrence and those without showed no significant differences regarding patients’ age, gender, or BMI, but there were significantly higher rates of patients with ASA ≥ 3 in the recurrence group (70% vs. 36.7%, *p* = 0.032). There were significantly more patients in the recurrence group who underwent a standard TAE (transanal excision) and not MIS resection compared with the patients with no recurrence (28.6% and 3.6%, respectively, *p* = 0.008). There was no significant difference in distance from the anal verge, the rate of full-thickness resection, the rate of rectal wall defect closure, the rate of intraoperative or postoperative complications, or length of hospital stay. When comparing the pathological reports, the recurrence group had larger tumors with a mean size of 4.3 ± 2.4 cm compared to 2.5 ± 1.2 cm in the non-recurrence group, *p* < 0.001, with a higher rate of tumors larger than 3.5 cm (61.5% vs. 17.1%). There was also a higher rate of involved margins in the recurrence group (41.7% vs. 6.2%, *p* = 0.003) and more mucinous adenocarcinomas (14.3% vs. 1.2%, *p* = 0.004). There were no differences between the groups regarding lymphovascular invasion, and tumor budding (Table 1).

On a multivariable logistic regression model, each pathological characteristic was tested in its own model while adjusted to the patient’s age. The most significant association and possible risk factors for cancerous recurrence were involved margins (OR = 9.59, CI—2.14–43.07, *p* = 0.003), having a tumor larger than 3.5 cm (OR = 6.67, CI—1.82–24.36, *p* = 0.004), and mucinous adenocarcinoma (OR = 14.02, CI—1.13–173.85, *p* = 0.04). Lymphovascular invasion and tumor budding were not found to be associated with recurrence (Table 5).

## 4. Discussion

This study demonstrates real-world experience with local excision of early rectal T1 tumors over eleven years in six academic centers. Following local excision of the T1 lesions, a quarter of patients went back for another surgery or added treatment, mainly proctectomy, due to involved margins, poor histology, or high-risk features. Although a T1 level of penetration of rectal cancer is considered the most favorable stage, this study showed a substantial rate of rectal cancer recurrence of almost 15%, mainly local recurrence at a mean interval of just over a year. Similar studies on local excision of T1 lesions have shown local recurrence rates from 4.1% to 20.5% [5,6,7,8,9], and 2.6–9.1% for distance metastasis recurrence [13,18,19]. The reported recurrence rates after formal proctectomy with a total mesorectal excision (TME) for T1 are 1.6–2% [8,20]. Although TME has a much higher potential advantage to avoid recurrence, there are much higher added morbidity and complication risks with TME compared to LE.

Certain risk factors should be addressed when trying to balance the potential elevated recurrence risk difference between LE and TME and the added morbidity with TME. The NCCN guidelines for LE in rectal cancer recommend LE for cases where the lesions take up less than 30% circumference of the rectum, are under 3 cm in size, mobile, nonfixed, and well to moderately differentiated, and there is no evidence of lymphadenopathy on pretreatment imaging [21]. Yet, the recurrence rate after LE of a T1 lesion is still higher than with a TME. This study attempted to identify risk factors that warrant special attention. As shown in a previous study and in this current one, not all patients with rectal polyps for LE undergo preoperative pelvic-specific imaging [22]. In this study, less than two-thirds underwent TRUS, and only 8% had a pelvic MRI. Although pelvis-specific imaging, TRUS, or MRI for every suspected rectal polyp with malignant biopsy are highly recommended in a preoperative setting [23,24], this study had insufficient data to prove this concept.

It has been shown that LE with an MIS approach is superior to the standard open TAE approach regarding complications, specimen quality, and recurrence rates [25]. Similar outcomes were seen in this study. TAE was performed for only low rectal lesions, had higher rates of involved margins, lower rates of full-thickness resection, had higher recurrence rates, and led to added surgery after pathology compared to the TEM and TAMIS approaches. The univariate analysis identified TAE as associated with recurrence and a possible risk factor, and should be avoided when possible for these kinds of lesions.

A quarter of patients went back for additional surgery or other treatment like chemotherapy or radiation following the initial local excision. It was unclear what was the main reason for going back to surgery, but when reviewing the pathological reports, eight patients had tumors larger than 3.5 cm, seven had involved margins, four had lymphovascular invasion, four had tumor budding, and one had mucinous pathology. Involved resection margins are clearly a risk factor for recurrence or ongoing disease, and as such, six out of ten patients with involved margins went back for another rectal resection. One might consider an involved margin in the local excision of a T1 lesion as a so-called ‘excisional biopsy’, and the added proctectomy as the ‘actual treatment’. Yet, these patients still showed a higher risk of recurrence nonetheless [26]. The time interval from LE to recurrence was on average thirteen months, and the overall mortality rate was 11% at almost six years from the initial diagnosis. Although data are lacking on disease-free survival and disease-specific mortality, these current data imply a very good prognosis for these patients.

The univariate and multivariate logistic regression analyses showed that the strongest and most significant risk factors for recurrence after local excision of T1 lesions were involved margins, the size of the tumor (mainly larger than 3.5 cm), a mucinous histology tumor, and to a lesser extent a transanal excision that is not MIS. Other pathological markers that were suggested as risk factors, like lymphovascular invasion, tumor budding, or the depth of resection, were not found to support this claim. The limited reported data in the literature on the comparison of local recurrence after LE with SM3 lesion compared to SM1-2 demonstrate rates of 30% vs. 0–16%, respectively [11]. Our data on SM involvement were limited to only a third of the cohort, and although it was not significant, it showed a strong trend toward an almost three times higher overall recurrence rate among SM3 cases, as high as 20%.

The limitations of this study revolve around its retrospective nature, which may lead to bias by interpreting casual associations as risk factors, and the relatively small sample size. As MIS LE evolved and came into practice, earlier adoption of these techniques might have changed the specimens’ quality and outcomes. Adding to that, more detailed pathology reports including SM involvement for all patients would have added power to the data. The multicentric nature of the study is considered an advantage but might also allow for differences in patient selection and treatment choice that might have influenced the results. Finally, answering why an early rectal tumor with a proper resection has a cancerous recurrence is still challenging, despite the risk factors we identified. Future exploration into the genetic makeshift of these tumors may offer a solution that we intend to follow.

## 5. Conclusions

Local excision of T1 rectal cancer is a safe and viable option but may carry an almost 25% risk for additional treatment. The risk for recurrence is nearly 15%, and the most prominent risk factors for recurrence are involved margins, tumors larger than 3.5 cm, mucinous histology, and to a lesser degree, the TAE approach and an SM3 involvement. These high-risk patients might warrant additional intervention and stricter surveillance protocols.

## Figures and Tables

**Table 1:** Baseline demographics, operative platform, postoperative complications, surgical and pathological findings in the overall T1 cohort, and a comparison between patients with and without recurrence.

Characteristic	Overall T1 (n = 97)	No Recurrence n = 83 (85.6%)	Recurrence n = 14 (14.4%)	*p*-Value
Age (year)—mean ± SD	68.4 ± 11	67.3 ± 10.8	74.5 ± 10	0.023
Female—n (%)	42 (43.3)	33 (39.8)	9 (64.3)	0.087
BMI (kg/m²)—mean ± SD	28 ± 4.8	27.7 ± 4.59	31.5 ± 6.5	0.046
ASA ≥ 3—n (%)	29 (36.7)	22 (31.9)	7 (70)	0.032
Operative platform				0.008
TAE—n (%)	7 (7.2)	3 (3.6)	4 (28.6)	
TAMIS/TEM—n (%)	90 (92.8)	80 (96.4)	10 (71.4)	
Distance from anal verge (cm)—mean ± SD	7.8 ± 3.2	7.95 ± 3.2	6.6 ± 3.1	0.169
Depth of resection				0.792
Full thickness—n (%)	82 (84.5)	70 (84.3)	12 (85.7)	
Partial thickness—n (%)	11 (11.3)	10 (12)	1 (7.1)	
Piecemeal—n (%)	4 (4.1)	3 (3.6)	1 (7.1)	
Rectal wall defect left open—n (%)	14 (14.4)	13 (15.7)	1 (7.1)	0.685
In-op complications—n (%)	8 (8.2)	6 (7.2)	2 (14.3)	0.325
Post-op complications—n (%)	8 (8.2)	6 (7)	2 (14.3)	0.325
LOS (days)—mean ± SD	3.4 ± 3.8	3.5 ± 4.1	2.8 ± 1.2	0.487
Pathological findings				
Tumor size (cm)—mean ± SD	2.7 ± 1.6	2.5 ± 1.2	4.3 ± 2.4	<0.001
Tumors > 3.5 cm—n (%)	22 (23.2)	14 (17.1)	8 (61.5)	0.002
Involved margins	10 (10.8)	5 (6.2)	5 (41.7)	0.003
Pathology grading				0.004
Well-differentiated—n (%)	50 (52.1)	39 (47.6)	11 (78.6)	
Mod-differentiated—n (%)	33 (34.4)	33 (40.2)	0	
Poor-differentiated—n (%)	5 (5.2)	5 (5.9)	0	
Mucinous Ca—n (%)	3 (3.1)	1 (1.2)	2 (14.3)	
Lymphovascular invasion—n (%)	9 (9.4)	8 (9.8)	1 (7.1)	1.000
Tumor budding—n (%)	12 (12.4)	10 (12.0)	2 (14.3)	0.683
Added treatment after pathology				0.673
LAR—n (%)	6 (6.3)	4 (4.9)	2 (14.3)	
APR—n (%)	8 (8.3)	7 (8.5)	1 (7.1)	
Redo local excision—n (%)	6 (6.3)	5 (6.1)	1 (7.1)	
Adjuvant therapy—n (%)	4 (4.2)	3 (3.7)	1 (7.1)	
Overall recurrence	14 (14.4%)	-	14 (14.4%)	
Local recurrence	11 (11.3%)	-	11 (11.3%)	
Systemic recurrence	6 (6.2%)	-	6 (6.2%)	
Interval time surgery to recurrence (years)—mean ± SD	1.1 ± 0.6	-	1.1 ± 0.6	

BMI—body mass index. ASA—American Society of Anesthesiologists score. TAE—transanal excision. TAMIS—transanal minimally invasive surgery. TEM—transanal endoscopic microsurgery. In-op—intraoperative. LOS—length of stay. LAR—low anterior resection. APR—abdominoperineal resection. Adjuvant therapy—radio/chemo/immune therapy.

**Table 2:** Operative platforms compared in quality of resection.

Characteristic	TAE (n = 7)	TAMIS (n = 29)	TEM (n = 61)	*p*-Value
Tumor size (cm)—mean ± SD	3.7 ± 2.6	2.8 ± 1.6	2.7 ± 1.5	0.137
Distance from anal verge (cm)—mean ± SD	4.6 ± 1.3	7.7 ± 2.9	7.8 ± 3.1	0.047
Margins				
Clear margins >3 mm—n (%)	3 (43)	27 (93)	40 (65.6)	0.046
Clear margins <3 mm—n (%)	1 (14)	1 (3.4)	11 (18)	
Involved margins—n (%)	2 (28.6)	2 (3.4)	7 (11.5)	
Missing data—n (%)	1 (14.3)	0	3 (4.9)	
Depth of resection				0.443
Full-thickness resection	5 (71.4)	27 (93.1)	50 (82)	
Partial-thickness resection	1 (14.3)	1 (3.4)	9 (14.8)	
Piecemeal	1 (14.3)	1 (3.4)	2 (3.3)	
Postoperative complications—n (%)	3 (43%)	3 (10%)	2 (3%)	0.001

TEA—transanal excision. TAMIS—transanal minimally invasive surgery. TEM—transanal endoscopic microsurgery.

**Table 3:** Added treatment after T1 pathology—characteristics.

Characteristic	LAR or APR (n = 14)	Re-do LE (n = 6)	Adjuvant therapy (n = 4)
Age—mean ± SD	65.4 ± 6.4	67.1 ± 10	78.7 ± 9.6
No. tumors >3.5 cm—n (%)	5 (35.7)	1 (16.7)	2 (50)
Involved margins—n (%)	3 (21.4)	3 (50)	1 (33.3)
Mucinous Adenocarcinoma—n (%)	0	0	1 (25)
Lymphovascular invasion—n (%)	4 (30.8)	0	0
Tumor budding—n (%)	3 (21.4)	0	1 (25)
Recurrence after added treatment			
Any recurrence—n (%)	3/14 (21.4)	1/6 (16.7)	1/4 (25)
Local recurrence—n (%)	2/14 (14.3)	1/6 (16.7)	1/4 (25)
Systemic recurrence—n (%)	2/14 (14.3)	0	0

LAR—low anterior resection. APR—abdominoperineal resection. LE—local excision. Adjuvant therapy—radio/ chemo/ immune therapy.

**Table 4:** Characteristics comparison according to submucosal involvement (SM), recurrence rates, and added treatment.

Characteristic	SM1 + SM2 (n = 14)	SM3 (n = 15)	*p* Value
Tumor size (cm)—mean ± SD	2.4 ± 1.2	3.5 ± 1.7	0.051
No. tumors > 3.5 cm—n (%)	2 (14.3)	6 (40)	0.215
Involved margins—n (%)	1 (7.1)	2 (15.4)	0.596
Mucinous adenocarcinoma—n (%)	0	1 (6.7)	1.000
Lymphovascular invasion—n (%)	0	1 (6.7)	1.00
Tumor budding—n (%)	2 (14.3)	3 (20)	1.000
Added treatment after pathology			0.366
LAR—n (%)	1 (7.1)	1 (6.7)	
APR—n (%)	1 (7.1)	2 (13.3)	
Redo local excision—n (%)	2 (14.3)	0	
Adjuvant therapy—n (%)	0	2 (13.3)	
Overall recurrence—n (%)	1 (7.1)	3 (20)	0.598
Local recurrence—n (%)	1 (7.1)	2 (13.3)	1.000
Systemic recurrence—n (%)	0	1 (6.7)	1.000
Mortality	2 (14.3)	4 (26.7)	0.651
Interval time from surgery to mortality (years)—mean ± SD	6 ± 4.5	2 ± 0.8	0.426
Follow-up time (years)—mean ± SD	3.3 ± 1.7	2.2 ± 1.7	0.087

LAR—low anterior resection. APR—abdominoperineal resection. Adjuvant therapy—radio/chemo/immune therapy.

**Table 5:** Multivariable analysis of risk factors for recurrence, adjusted to age.

Characteristic	OR	CI (95%)	*p* Value
Involved margins	9.59	2.14–43.07	0.003
Tumor size > 3.5 cm	6.67	1.82–24.36	0.004
Mucinous adenocarcinoma	14.02	1.13–173.85	0.040
Lymphovascular invasion	1.287	0.13–12.47	0.828
Tumor budding	1.427	0.26–7.71	0.769

## Data Availability

Data will be available upon request.
